# Calcium-binding S100 protein expression in pterygium

**Published:** 2009-02-16

**Authors:** Andri K. Riau, Tina T. Wong, Roger W. Beuerman, Louis Tong

**Affiliations:** 1 Singapore Eye Research Institute, Singapore; 2 Singapore National Eye Center, Singapore; 3 Department of Ophthalmology, Yong Loo Lin School of Medicine, National University of Singapore, Singapore

## Abstract

**Purpose:**

Pterygium is an ocular surface disease of unknown etiology associated with epithelial and fibrovascular outgrowth from the conjunctiva onto the cornea. S100 proteins are calcium-activated signaling proteins that interact with other proteins to modulate biological functions such as cell migration, proliferation, and differentiation. The aim of this study was to investigate the presence of various S100 proteins in pterygium compared to normal conjunctiva.

**Methods:**

Immunofluorescent staining using antibodies against S100A4, S100A6, S100A8, S100A9, and S100A11 were conducted to investigate the expression and tissue distribution. S100 protein secretions and expressions were confirmed using western blot and quantitative real-time polymerase chain reaction (RT-PCR), respectively.

**Results:**

Immunofluorescent staining demonstrated the presence of S100A4, S100A6, S100A8, S100A8, S100A9, and S100A11 in both conjunctival and pterygial epithelium. No significant difference was found in the localization and expression of S100A4. In both conjunctiva and pterygium, S100A4-positive cells were found in superficial and suprabasal layers. S100A6 expression was strong in the superficial layer of pterygium epithelium but relatively weaker in the suprabasal and superficial cells of normal conjunctiva epithelium. S100A8 and S100A9 were localized in the superficial layer of both pterygium and normal conjunctiva epithelium, with higher levels in pterygium than uninvolved conjunctiva. S100A11 was expressed in the basal cells of conjunctival epithelium but in the suprabasal layers of pterygium epithelium. Western blot and RT–PCR confirmed the presence of S100A4, S100A6, S100A8, S100A9, and S100A11 in pterygium and conjunctiva tissue.

**Conclusions:**

Higher levels of S100A6, S100A8, and S100A9 expressions were detected in the pterygium tissue relative to normal conjunctiva. In addition, a distinct alteration of localization of S100A11 expression was observed in pterygium epithelium compared to the conjunctiva. Therefore, these S100 proteins may be associated with the formation of pterygium.

## Introduction

Pterygium is a common ocular surface disease characterized by epithelial and fibrovascular outgrowth of conjunctiva over the cornea. The wing-shaped epithelium of pterygium that invades the cornea centripetally displays squamous metaplasia and goblet cell hyperplasia [1]. On many occasions, pterygium can impair visual function in instances such as irregular astigmatism or impaired tear film regularity induced by pterygium and visual occlusion induced by a large pterygium that has migrated to the visual axis over the central cornea. Many etiologic factors have been described for this disease. Compelling evidence that ultraviolet (UV)-mediated limbal damage triggers this pathogenesis has been studied extensively by Coroneo et al. [2,3]. Other causes include an anomaly in epidermal proliferation [4], inflammation [5], stem cell dysfunction [6], changes in extracellular matrix [7], metabolic disorder [8], neuronal dysfunction [9], and alterations in epithelial-mesenchymal transition [10].

S100 proteins comprise a multitude of low molecular weight, calcium-binding proteins that interact with other proteins to modulate biological processes [11]. They have been named S100 due to their biochemical property of remaining soluble after precipitation with 100% ammonium sulfate [12]. Thirteen members of the family are clustered within the epidermal differentiation complex located on chromosome 1q21 [13]. This region is of particular interest because it encodes genes that are expressed in epidermal keratinocytes such as involucrin, filaggrin, repetin, and trichohyalin [14], which are also expressed on the ocular surface [15]. S100 is characterized by the presence of two calcium binding sites of the EF-hand type (helix–loop–helix), one of which is located in the NH_2_-terminal and is non-canonical while the other binding site is located in the COOH-terminal and is canonical [16]. This configuration enables S100 proteins to respond to a calcium stimulus induced by cell signaling.

Possible S100 protein functions in keratinocytes have been reviewed in a previous article [17]. One of them is acting as a chemotactic agent. Kerkhoff et al. [18] have showed that S100A8 and S100A9 are released from neutrophils by a microtubule-dependent mechanism and may induce inflammation by influencing leukocyte trafficking. In addition, S100A8 and S100A9 released from keratinocytes may initiate immune cell invasion, which can be further propagated by the release of S100A8 and S100A9 from incoming neutrophils [19]. S100 proteins were proposed to facilitate the membrane remodeling process as well, which is evident by the interaction of S100A11 with membrane lipids to join different segments of the membrane or bend the membrane surface [17]. S100A11 is also a cross-linked component of the cornified envelope, a structure that is assembled from membrane-associated constituents [20]. Recently, S100A4, S100A6, S100A8, and S100A9 were also shown to have potential roles in the wound healing mechanism [19,21-23]. Chemotaxis, tissue remodeling and wound healing defects, and anomaly in cornified envelope assembly are some of the proposed mechanisms in pterygium formation [1,9,15,24-26].The biological processes regulated by S100 proteins appear to be implicated in pterygium formation as well. Therefore, we proceeded to investigate S100 expression in pterygium. To the best of our knowledge, there has not been a study on the expression profile, tissue and subcellular localization, and function of S100 proteins in the human ocular surface and pterygium. Thus, we conducted a study to investigate the pattern of expression of several S100 proteins (S100A4, S100A6, S100A8, S100A9, and S100A11) in uninvolved conjunctival and pterygium tissues. Our study also aimed to identify the specific changes in the localization and expression levels of the S100 proteins in pterygium relative to the normal conjunctiva.

## Methods

### Patients and specimens

The method of patient specimen collection was performed as previously described [27,28]. Briefly, the pterygium tissue from a patient was compared with the uninvolved conjunctival tissue from the same eye that was excised from the superior temporal quadrant of the bulbar conjunctiva, next to the position of the harvested free conjunctival graft. Paired tissues were obtained from 12 patients. All protocols adhered to the tenets of the Declaration of Helsinki and were approved by the institutional review board of the Singapore Eye Research Institute. Written informed consents were acquired from all participating patients.

### Materials and reagents

Mouse monoclonal antibodies against S100A4, S100A6, S100A9, and S100A11 were purchased from Abnova (Taipei, Taiwan), and mouse monoclonal antibody against S100A8 from Acris Antibodies (Hiddenhausen, Germany) was used. Goat anti-mouse HRP-conjugated antibody, radio immunoprecipitation (RIPA) lysis buffer, and UltraCruz Mounting Medium containing 4.6-diamidino-2-phenylindole (DAPI) were purchased from Santa Cruz Biotechnology (Santa Cruz, CA). Goat anti-mouse Alexa Fluor 488 was purchased from Invitrogen (Carlsbad, California). The Universal ProbeLibrary set was from Roche Applied Science (Mannheim, Germany). Stock PBS (10X) was purchased from 1st Base (Singapore). Bovine serum albumin (BSA), Tween-20, and Triton X-100 were from Sigma (St. Louis, MO). Nitrocellulose membrane and non-fat milk were purchased from Bio-Rad Laboratories (Hercules, CA). Super Signal West Pico chemiluminescence reagent was from Pierce Biotechnology (Rockford, IL).

### Immunofluorescent staining

Conjunctival tissues from normal conjunctival epithelium and pterygium were sectioned with a Microm HM550 cryostat (Microm, Walldorf, Germany) at 6 μm thickness. Sections were fixed with ice-cold acetone for 15 min, washed with 1X PBS, blocked with 4% BSA in 1X PBS containing 0.1% Triton X-100 (Sigma) for 1 h, and then incubated with primary antibodies (Table 1) diluted in the blocking solution at 4 °C overnight. After washing with 1X PBS, the sections were incubated with Alexa Fluor 488-conjugated secondary antibody at room temperature for 1 h. Slides were then mounted with UltraCruz Mounting Medium. For negative controls, non-immune serum was used in place of the specific primary antibody. Sections were observed under and imaged with a Zeiss Axioplan 2 fluorescence microscope (Zeiss, Oberkochen, Germany).

**Table 1:** Primary antibodies used in the experiment.

**Target antigen**	**Category (clone)**	**Manufacturer**	**Dilution factor**	
			**IF**	**WB**
S100A4	Mouse monoclonal (1F12–1G7)	Abnova	1/200	1/500
S100A6	Mouse monoclonal (6B5)	Abnova	1/300	1/1000
S100A8	Mouse monoclonal (8–5C2)	Acris Antibodies	1/150	1/1000
S100A9	Mouse monoclonal (1C10)	Abnova	1/150	1/1000
S100A11	Mouse monoclonal (2F4)	Abnova	1/150	1/1000

This table describes the primary antibodies used in the western blots and immunoflourescent staining experiments. The respective animal host, manufacturer, and dilution factor used in the experiments were displayed. IF, immunofluorescent staining; WB, western blot.

### Western blot

Normal conjunctiva and pterygium tissue were homogenized individually into RIPA lysis buffer. Insoluble materials were removed by 15 min centrifugation (10,000x g) at 4 °C. An equal volume of 4X SDS loading buffer was added to each sample, which was then subjected to boiling for 5 min at 99 °C. The sample was then left on ice for 10 min before reduction on a sodium dodecyl sulfate-polyacrylamide gel electrophoresis (15% SDS–PAGE gradient). The amounts of protein applied was 60 µg. Resolved proteins were electrophoretically transferred onto a nitrocellulose membrane and blocked in 1X PBS containing 5% non-fat milk followed by overnight incubation with the primary antibody (Table 1) at room temperature with agitation. The membranes were then washed vigorously three times each for 5 min in 1X PBS and 0.1% Tween-20. The goat anti-mouse HRP-conjugated secondary antibody was then applied at a dilution of 1:2000. Immunoreactivity was visualized with Super Signal West Pico chemiluminescence reagent.

### Real-time polymerase chain reaction 

Table 2 shows the primers tested for the detection of various S100 transcripts. Reverse transcription of 0.5 μg of RNA for each sample was performed as previously described [15]. RT–polymerase chain reaction (PCR) was performed by using the Lightcycler 480 System (Roche Diagnostics, Basel, Switzerland). For each reaction, the appropriate pre-synthesized hydrolysis FAM (excitation wavelength of 483-533 nm) probe was selected from the Universal ProbeLibrary set, based on the ProbeFinder web-based assay design tool selecting for intron spanning assays. Glyceraldehyde-3-phosphate dehydrogenase (*GAPDH*) was used as the internal control. For each pair of primers and samples, triplicate wells were used. ΔC_t_ was calculated by subtracting the C_t_ of *GAPDH* from the C_t_ of the targeted gene. The un-involved conjunctiva sample was considered the calibrator to compare the relative abundance of each tested *S100* gene transcript in the pterygium sample. The fold change was determined by the equation, 2^(-ΔΔCt)^, where ΔΔCt=ΔCt_sample_−ΔCt_calibrator_. A probability level of p<0.05 was considered statistically significant.

**Table 2:** Primers used in real-time PCR.

**Gene**	**NCBI accession number**	**Primer sequence**
*S100A4*	NM_002961.2	F: CGCTTCTTCTTTCTTGGTTTG
		R: GAGTACTTGTGGAAGGTGGACA
*S100A6*	NM_014624.3	F: ACTGCGACACAGCCCATC
		R: GAAGATGGCCACGAGGAG
*S100A8*	NM_002964.3	F: CAAGTCCGTGGGCATCAT
		R: GACGTCGATGATAGAGTTCAAGG
*S100A9*	NM_002965.3	F: GTGCGAAAAGATCTGCAAAA
		R: TCAGCTGCTTGTCTGCATTT
*S100A11*	NM_005620.1	F: TGAGGAGAGGCTCCAGACC
		R: ACCGCTCAGTCTCTGTAGGG
*GAPDH*	AF261085.1	F: AGCCACATCGCTGAGACA
		R: GCCCAATACGACCAAATCC

This table shows the various primers used against the S100 protein genes and *GADPH* in the polymerase chain reactions. The NCBI accession numbers of the sequences used to design the forward or 5' primer (F) and reverse or 3' primer (R) were shown, in a 5' to 3' orientation.

## Results

### S100 protein localization and expression

**Figure 1:**
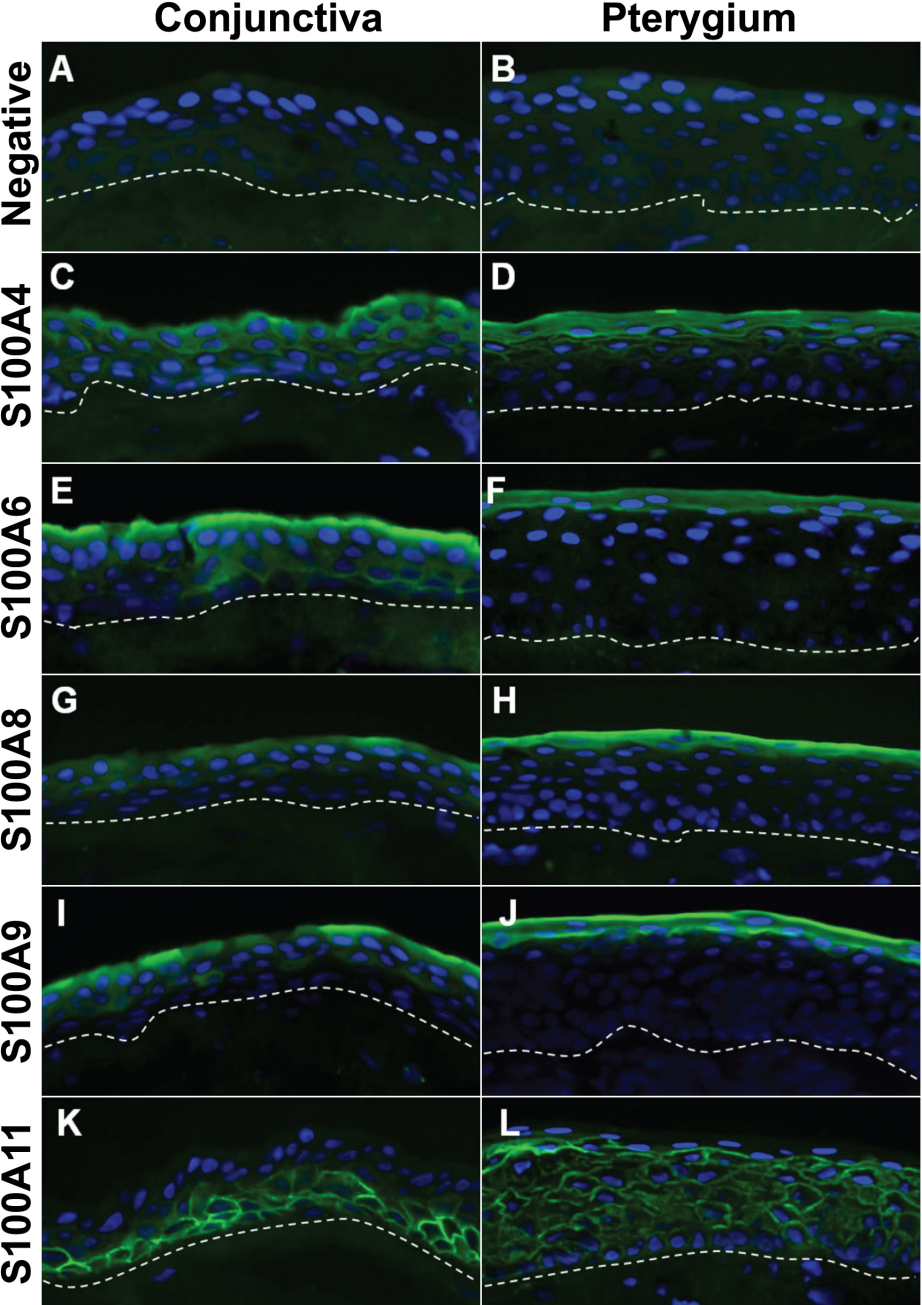
Immunofluorescent staining of S100 proteins in human conjunctival and pterygial epithelia. Primary antibodies against S100A4 (**C** and **D**), S100A6 (**E** and **F**), S100A8 (**G** and **H**), S100A9 (**I** and **J**), and S100A11 (**K** and **L**) were used. Negative controls were shown in **A** and **B**. Images of uninvolved conjunctiva (**A**, **C**, **E**, **G**, **I**, and **K**) and pterygium epithelium (**B**, **D**, **F**, **H**, **J**, and **L**) were shown. The nuclei were stained with DAPI present in the mounting medium. A dashed line indicates the location of the basement membrane. All images were taken at 400X magnification.

Immunofluorescent staining detected the presence of S100A4, S100A6, S100A8, S100A9, and S100A11 in normal human conjunctival and pterygium epithelia. No fluorescent signals were detected in the negative controls (Figure 1A,B). S100A4 expression was found in the superficial and suprabasal layers of both the normal conjunctival (Figure 1C) and pterygium (Figure 1D) epithelia. The expression was localized in the plasma membrane and cytoplasm of the superficial cells and in the membrane of the suprabasal cells of both epithelia.

S100A6 was detected in the membrane and cytoplasm of the superficial epithelial cells as well as some suprabasal cells of normal conjunctival epithelium (Figure 1E) but was found only in the superficial layer of pterygium epithelium (Figure 1F). A similar staining pattern was also observed with the expression of S100A8 and S100A9 in the uninvolved conjunctiva and pterygium (Figures 1G,I). A distinctly different expression pattern of S100A11 was noted in pterygium epithelium when compared to the uninvolved conjunctiva. S100A11 was stained in the plasma membrane of the basal layer of normal conjunctival epithelium (Figure 1K) while staining was more prominent in the suprabasal layer of the pterygium tissue (Figure 1L).

**Figure 2:**
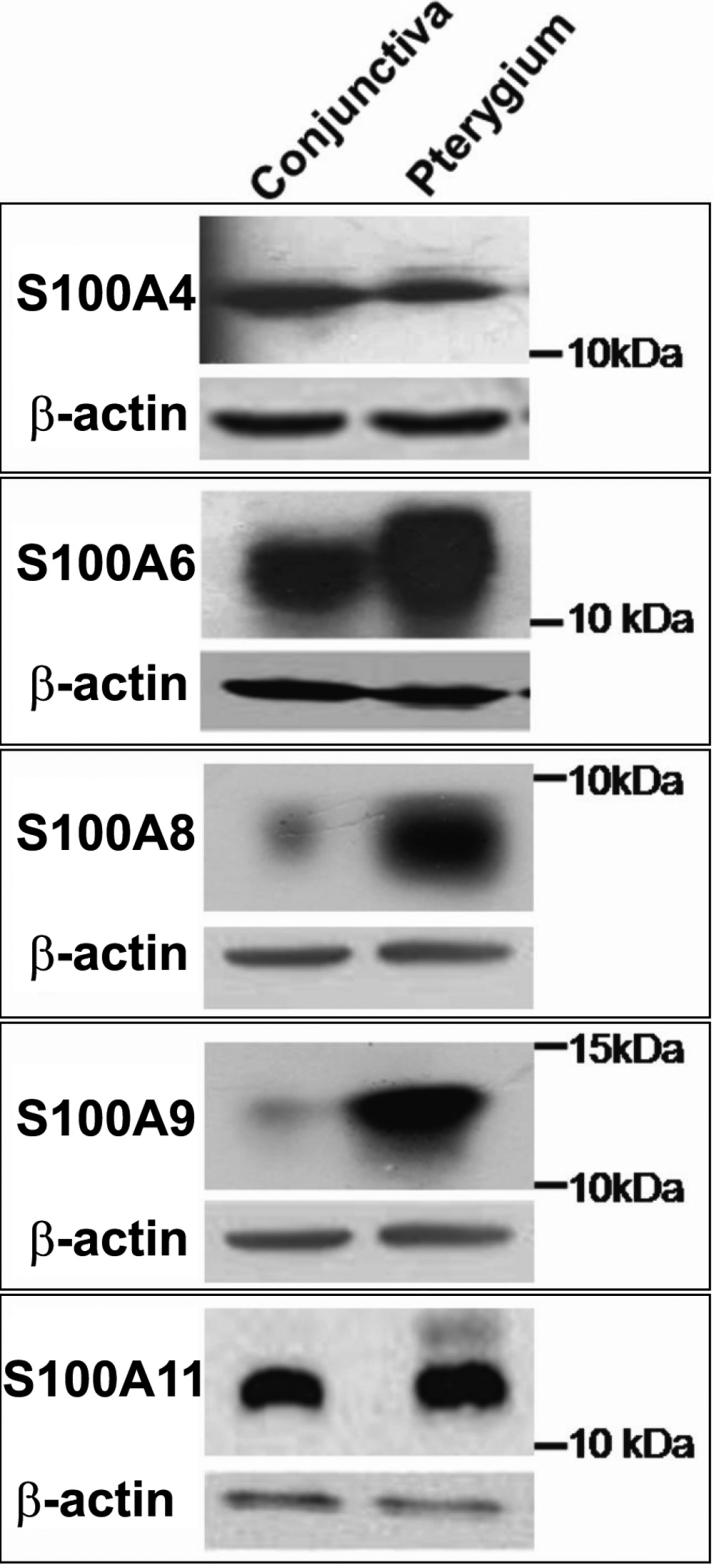
Western blot analysis of S100 proteins expressed in human conjunctiva and pterygium tissue. Uninvolved conjunctiva (Lane 1) and pterygium (Lane 2) tissues were lysed in RIPA buffer and 60 μg of total protein was loaded in each lane of SDS-PAGE and transferred to nitrocellulose membrane and probed with antibodies against various S100 proteins as well as β-actin (loading control). Bands of 11 kDa corresponding to S100A4, S100A6, and S100A11 were detected. Bands corresponding to the molecular weight of 8 kDa and 13 kDa confirmed the presence of S100A8 and S100A9, respectively.

Western blot (Figure 2) confirmed the presence of the tested S100 proteins in human normal conjunctiva and pterygium tissue. Immunoreactive bands corresponding to molecular weight of approximately 11 kDa showed the presence of S100A4, S100A6, and S100A11. Protein levels of S100A4 and S100A11 in the pterygium tissue was not significantly different compared with the normal conjunctiva. However, expression of S100A6, S100A8, and S100A9 were markedly greater in the pterygium tissue compared to the uninvolved conjunctival tissue.

### S100 transcript level

**Figure 3:**
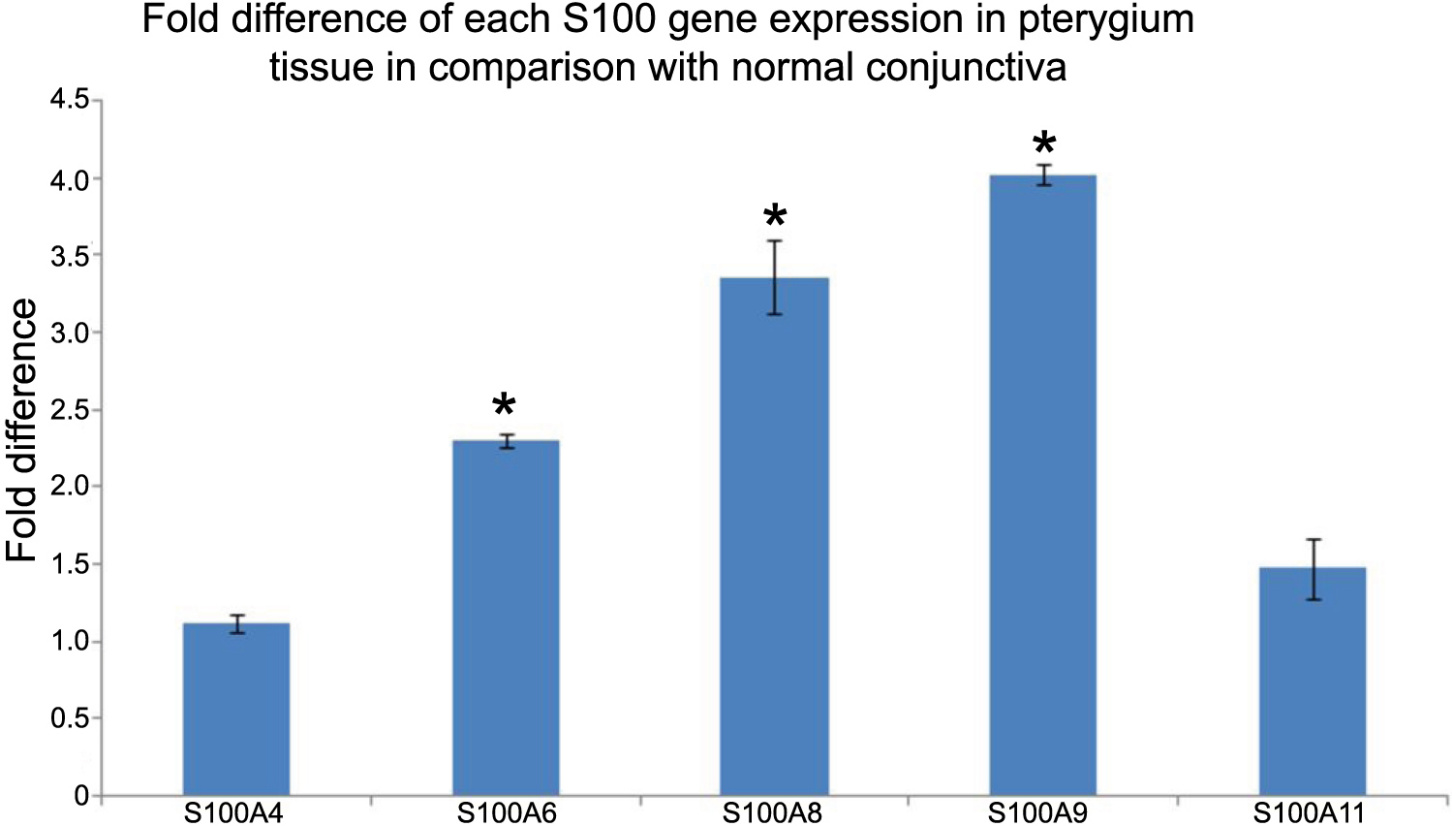
Fold difference of *S100A4*, *S100A6*, *S100A8*, *S100A9*, and *S100A11* gene transcripts in pterygium tissue relative to conjunctiva tissue. Total RNA was extracted from uninvolved conjunctiva and pterygium tissue after mechanical homogenization. Reverse transcription for 0.5 μg of total RNA. Glyceraldehyde-3-phosphate dehydrogenase (*GAPDH*) was used as the internal control. ΔC_t_ was calculated by subtracting the C_t_ of *GAPDH* from the C_t_ of the targeted gene. The fold change was determined by the ΔΔC_t_ method (see text). The uninvolved conjunctiva sample was used as a reference for each of the tested S100 transcripts in the pterygium sample. Height of the bars represents mean value and error bars represent standard deviation. An asterisk indicates that p is less than 0.05 (p<0.05).

*S100A4*, *S100A6*, *S100A8*, *S100A9*, and *S100A11* gene transcripts were detected in conjunctiva and pterygium tissues. Similar to the protein expression, *S100A4* and *S100A11* did not demonstrate any significant difference in expression in either pterygium or conjunctival tissues. However, *S100A6*, *S100A8*, and *S100A9* transcripts were upregulated (p<0.05) in pterygium relative to normal conjunctiva. This result corroborated with the protein expression. In pterygium tissue, *S100A6* expression increased 2.30 fold, *S100A8* increased 3.36 fold, and *S100A9* increased 4.01 fold relative to normal conjunctiva. A bar graph summarizing the fold difference of these genes in pterygium tissue compared to the conjunctiva tissue is illustrated in Figure 3.

## Discussion

To the best of our knowledge, there have been no studies describing the expression profiles of S100 proteins in the normal conjunctiva and diseases associated with it. Our investigation revealed that S100A4, S100A6, S100A8, S100A9, and S100A11 are expressed in both the normal and pterygium epithelia. The role of various S100 proteins has been well documented in the normal skin epidermis and many cutaneous diseases and tumors [17,29-33]. One of the proposed causes for pathogenesis of pterygium is defective cell differentiation [34]. Several studies have elucidated a role for S100 proteins in epithelial differentiation [14,35,36]. S100A8 and S100A9, which form homo- and heterodimers and are frequently co-expressed [37], were shown in vivo and in vitro to bind to keratin filaments [35,36]. Keratin proteins are encoded by epidermal differentiation complex. Since these proteins are expressed during terminal differentiation and are associated with intermediate filaments, a functional role in the reorganization of the cytoskeleton during hyperproliferative ocular surface disease such as pterygium may be assumed for S100A8 and S100A9. Our findings, which showed an increased expression of S100A8 and S100A9 in the superficial layer of pterygium epithelium, support the notion that S100 proteins are centrally involved in cell maturation processes.

We found the presence of S100A11 in the plasma membrane of uninvolved human conjunctiva and pterygium epithelial cells. This is consistent with the localization of S100A11 in the cell periphery during keratinocyte differentiation that has previously been described in vivo [30] and in vitro [38]. As part of the differentiation process, the individual S100A11 molecules have been known to be covalently cross-linked to form the cornified envelope [20]. Menon et al. [39] reported a gradient of increasing free calcium concentration as cells move from the basal to suprabasal layers during epidermal differentiation. A similar pattern of alteration in the localization of S100A11 was seen in our study, which further suggests that pterygium is related to an anomaly in epithelial differentiation.

A complex model of S100A11 interaction with annexin I has been proposed to explain the mechanism of action [40]. The S100A11/annexin I complex in the plasma membrane creates a calcium channel that permits calcium influx in terminally differentiated keratinocytes [40]. The increased intracellular calcium may activate transglutaminase (TG) 1, the enzyme responsible for the cornified envelope assembly in stratified epithelium [8]. This relationship suggests that S100A11 may function to facilitate membrane remodeling during terminal differentiation in the ocular surface. Future studies will investigate the colocalization and interactions of S100A11, annexin I, and TG on the ocular surface.

Pterygium can be considered a wound healing defect [10]. TG 2 has been known to play a crucial role in wound healing, cell migration, apoptosis, and maintenance of ocular surface integrity [41]. The distribution of this enzyme in pterygium has been investigated by Kim et al. [25]. Recently, it was reported that S100A11 is also a TG 2 substrate [42]. In addition, S100A4 has been linked with corneal fibroblast regeneration [21] and S100A6 with the corneal wound healing [22]. These findings suggest a potential role for S100 proteins in epithelial wound repair and response to stress in the formation of pterygium.

Another process in pterygium pathogenesis is abnormal fibrovascular and epithelial proliferation [4,7]. Pterygium has traditionally been considered a chronic degenerative condition. However, it can also be regarded as a benign lesion or tumor with abnormal p53 expression in the epithelium [43]. S100A4 plays a role in metastasis and cell proliferation and has been implicated in the development of epithelial tumors in the skin [28,44,45]. However, a high level of expression of these proteins was not found in other tumors, e.g., malignant melanoma [46] and esophageal squamous cell carcinoma [47]. Furthermore, we did not find any significant change in expression level of S100A4 in the pterygium relative to the conjunctiva, consistent with the lack of metastatic behavior in pterygium. Moreover, limbal stem cell dysfunction was proposed as one of the causes of pterygium [6]. Recently, Ito et al. [48] reported possible roles of S100A4 and S100A6 in the activation of stem cells at the onset of hair follicle regeneration. The differential localization of S100A4 and S100A6 demonstrated in our study raises the possibility of a stem cell defect in pterygium.

A limitation of this study was the small patient sample, although the findings were highly reproducible within these patients. Another limitation was that the uninvolved conjunctiva was excised from the superior temporal quadrant of the bulbar conjunctiva, next to the position of the harvested conjunctiva graft. Although this is far away from the site of pterygium, we accept that the conjunctiva may not be totally normal.

Several family members of S100 proteins have been used as tumor progression markers [49,50]. Recently, our laboratory detected a marked increase in the expression of S100A8 and S100A9 in the tear fluid of pterygium patients relative to the normal conjunctiva [51]. The findings in tissues reported here suggest that tear analysis can possibly detect pterygium progression. Therefore, possible detection of S100 protein levels may be useful as a clinical indicator for grading the progression as well as severity of pterygium. It remains to be seen whether targeting S100 proteins can help in the treatment of pterygium.

In summary, a change in level of expression was detected in S100A6, S100A8, and S100A9 in pterygium tissue relative to the normal conjunctiva. In addition, distinct alteration of the localization of S100A11 expression was observed in the pterygium epithelium compared to the normal conjunctiva. Therefore, these S100 proteins may be associated with pterygium pathogenesis. Our current study will be followed by an investigation of the functional implications of the S100 proteins in the ocular surface as well as their roles in pterygium.

## References

[1] Di Girolamo N, Chui J, Coroneo MT, Wakefield D (2004). Pathogenesis of pterygia: role of cytokines, growth factors, and matrix metalloproteinases.. Prog Retin Eye Res.

[2] Coroneo MT (1993). Pterygium as an early indicator of ultraviolet insolation: a hypothesis.. Br J Ophthalmol.

[3] Coroneo MT, Di Girolamo N, Wakefield D (1999). Pathogenesis of pterygia.. Curr Opin Ophthalmol.

[4] Tan DT, Liu YP, Sun L (2000). Flow cytometry measurements of DNA content in primary and recurrent pterygia.. Invest Ophthalmol Vis Sci.

[5] Di Girolamo N, Wakefield D, Coroneo MT (2006). UVB-mediated induction of cytokines and growth factors in pterygium epithelial cells involves cell surface receptors and intracellular signaling.. Invest Ophthalmol Vis Sci.

[6] Dushku N, Reid TW (1994). Immunohistochemical evidence that human pterygia originate from an invasion of vimentin-expressing altered limbal epithelial basal cells.. Curr Eye Res.

[7] John-Aryankalayil M, Dushku N, Jaworski CJ, Cox CA, Schultz G, Smith JA, Ramsey KE, Stephan DA, Freedman KA, Reid TW, Carper DA (2006). Microarray and protein analysis of human pterygium.. Mol Vis.

[8] Tong L, Li J, Chew J, Tan D, Beuerman R (2008). Phospholipase D in the human ocular surface and in pterygium.. Cornea.

[9] Chui J, Di Girolamo N, Coroneo MT, Wakefield D (2007). The role of substance P in the pathogenesis of pterygia.. Invest Ophthalmol Vis Sci.

[10] Chui J, Di Girolamo N, Coroneo MT, Wakefield D (2008). The pathogenesis of pterygium: Current concepts and their therapeutic implications.. Ocul Surf.

[11] Tu CL, Chang W, Bikle DD (2001). The extracellular calcium-sensing receptor is required for calcium-induced differentiation in human keratinocytes.. J Biol Chem.

[12] Volz A, Korge BP, Compton JG, Ziegler A, Steinert PM, Mischke D (1993). Physical mapping of a functional cluster of epidermal differentiation genes on chromosome 1q21.. Genomics.

[13] Moore BW (1965). A soluble protein characteristic of the nervous system.. Biochem Biophys Res Commun.

[14] Mischke D, Korge BP, Marenholz I, Volz A, Ziegler A (1996). Genes encoding structural proteins of epidermal cornification and S100 calcium-binding proteins form a gene complex (“epidermal differentiation complex”) on human chromosome 1q21.. J Invest Dermatol.

[15] Tong L, Corrales RM, Chen Z, Villarreal AL, De Paiva CS, Beuerman R, Li DQ, Pflugfelder SC (2006). Expression and regulation of cornified envelope proteins in human corneal epithelium.. Invest Ophthalmol Vis Sci.

[16] Donato R (2003). Intracellular and extracellular roles of S100 proteins.. Microsc Res Tech.

[17] Eckert RL, Broome AM, Ruse M, Robinson N, Ryan D, Lee K (2004). S100 proteins in the epidermis.. J Invest Dermatol.

[18] Kerkhoff C, Klempt M, Kaever V, Sorg C (1999). The two calcium-binding proteins, S100A8 and S100A9, are involved in the metabolism of arachidonic acid in human neutrophils.. J Biol Chem.

[19] Thorey IS, Roth J, Regenbogen J, Halle JP, Bittner M, Vogl T, Kaesler S, Bugnon P, Reitmaier B, Durka S, Graf A, Wockner M, Rieger N, Konstantinow A, Wolf E, Goppelt A, Werner S (2001). The Ca2+-binding proteins S100A8 and S100A9 are encoded by novel injury-regulated genes.. J Biol Chem.

[20] Robinson NA, Lapic S, Welter JF, Eckert RL (1997). S100A11, S100A10, annexin I, desmosomal proteins, small proline-rich proteins, plasminogen activator inhibitor-2, and involucrin are components of the cornified envelope of cultured human epidermal keratinocytes.. J Biol Chem.

[21] Ryan DG, Taliana L, Sun L, Wei ZG, Masur SK, Lavker RM (2003). Involvement of S100A4 in stromal fibroblasts of the regenerating cornea.. Invest Ophthalmol Vis Sci.

[22] Bazan HEP, Allan G, Bazan NG (1992). Enhanced expression of the growth-related calcyclin gene during corneal wound healing.. Exp Eye Res.

[23] Li X, Mohan S, Gu W, Miyakoshi N, Baylink DJ (2000). Differential protein profile in the ear-punched tissue of regeneration and non-regeneration strains of mice: a novel approach to explore the candidate genes for soft-tissue regeneration.. Biochim Biophys Acta.

[24] Tekelioglu Y, Turk A, Avunduk AM, Yulug E (2006). Flow cytometrical analysis of adhesion molecules, T-lymphocyte subpopulations and inflammatory markers in pterygium.. Ophthalmologica.

[25] Kim YJ, Park ES, Song KY, Park SC, Kim JC (1998). Glutathione transferase (class π) and tissue transglutaminase (Tgase C) expression in pterygia.. Korean J Ophthalmol.

[26] Di Girolamo N, Coroneo M, Wakefield D (2005). Epidermal growth factor receptor signaling is partially responsible for the increased matrix metalloproteinase-1 expression in ocular epithelial cells after UVB radiation.. Am J Pathol.

[27] Wong YW, Chew J, Yang H, Tan DT, Beuerman R (2006). Expression of insulin-like growth factor binding protein-3 in pterygium tissue.. Br J Ophthalmol.

[28] Tan DTH, Tan WY, Liu YP, Goh HS, Smith DR (2000). Apoptosis and apoptosis related gene expression in normal conjunctiva and pterygium.. Br J Ophthalmol.

[29] Gebhardt C, Nemeth J, Angel P, Hess J (2006). S100A8 and S100A9 in inflammation and cancer.. Biochem Pharmacol.

[30] Broome AM, Ryan D, Eckert RL (2003). S100 protein subcellular localization during epidermal differentiation and psoriasis.. J Histochem Cytochem.

[31] Shrestha P, Muramatsu Y, Kudeken W, Mori M, Takai Y, Ilg EC, Schafer BW, Heizmann CW (1998). Localization of Ca^2+^-binding S100 proteins in epithelial tumours of the skin.. Virchows Arch.

[32] Lesniak W, Slomnicki LP, Kuznicki J (2007). Epigenetic control of the S100A6 (calcyclin) gene expression.. J Invest Dermatol.

[33] Boni R, Burg G, Doguoglu A, Ilg EC, Schafer BW, Muller B, Heizmann CW (1997). Immunohistochemical localization of the Ca^2+^ binding S100 proteins in normal human skin and melanocytic lesions.. Br J Dermatol.

[34] Di Girolamo N, Tedla N, Kumar RK, McCluskey P, Lloyd A, Coroneo MT, Wakefield D (1999). Culture and characterization of epithelial cells from human pterygia.. Br J Ophthalmol.

[35] Goebeler M, Roth J, van den Bos C, Ader G, Sorg C (1995). Increase of calcium levels in epithelial cells induces translocation of calcium-binding proteins migration inhibitory factor-related protein 8 (MRP8) and MRP14 to keratin intermediate filaments.. Biochem J.

[36] Roth J, Burwinkel F, van den Bos C, Goebeler M, Vollmer E, Sorg C (1993). MRP8 and MRP14, S100-like proteins associated with myeloid differentiation, are translocated to plasma membrane and intermediate filaments in a calcium-dependent manner.. Blood.

[37] Teigelkamp S, Bhardwaj RS, Roth J, Meinardus-Hager G, Karas M, Sorg C (1991). Calcium-dependent complex assembly of the myeloic differentiation proteins MRP-8 and MRP-14.. J Biol Chem.

[38] Broome AM, Eckert RL (2004). Microtubule-dependent redistribution of a cytoplasmic cornified envelope precursor.. J Invest Dermatol.

[39] Menon GK, Grayson S, Elias PM (1985). Ionic calcium reservoirs in mammalian epidermis: Ultrastructural localization by ion-capture cytochemistry.. J Invest Dermatol.

[40] Rety S, Osterloh D, Arie JP, Tabaries S, Seeman J, Russo-Marie F, Gerke V, Lewit-Bentley A (2000). Structural basis of the Ca(2+)-dependent association between S100C (S100A11) and its target, the N-terminal part of annexin I.. Structure.

[41] Zhang W, Shiraishi A, Suzuki A, Zheng X, Kodama T, Ohashi Y (2004). Expression and distribution of tissue transglutaminase in normal and injured rat cornea.. Curr Eye Res.

[42] Ruse M, Lambert A, Robinson N, Ryan D, Shon KJ, Eckert RL (2001). S100A7, S100A10, and S100A11 are transglutaminase substrates.. Biochemistry.

[43] Tan DT, Lim AS, Goh HS, Smith DR (1997). Abnormal expression of the p53 tumor suppressor gene in the conjunctiva of patients with pterygium.. Am J Ophthalmol.

[44] Davies MPA, Rufland PS, Robertson L, Parry EW, Jolicoeur P, Barraclough R (1996). Expression of the calcium-binding protein S100A4 (p9Ka) in MMTV-neu transgenic mice induces metastasis of mammary tumours.. Oncogene.

[45] Ilg EC, Schafer BW, Heizmann CW (1996). Expression pattern of S100 calcium-binding proteins in human tumors.. Int J Cancer.

[46] Maelandsmo GM, Florenes VA, Mellingsaeter T, Hovig E, Kerbel RS, Fodstad O (1997). Differential expression patterns of S100A2, S100A4 and S100A6 during progression of human malignant melanoma.. Int J Cancer.

[47] Ji J, Zhao L, Wang X, Zhou C, Ding F, Su L, Zhang C, Mao X, Wu M, Liu Z (2004). Differential expression of S100 gene family in human esophageal squamous cell carcinoma.. J Cancer Res Clin Oncol.

[48] Ito M, Kizawa K (2001). Expression of calcium-binding S100 proteins A4 and A6 in regions of the epithelial sac associated with the onset of hair follicle regeneration.. J Invest Dermatol.

[49] Bolander A, Agnarsdottir M, Wagenius G, Stromberg S, Ponten F, Ekman S, Brattstrom D, Larsson A, Finarsson R, Ullenhag G, Hesselius P, Bergqvist M (2008). Serological and immunohistochemical analysis of S100 and new derivatives as markers for prognosis in patients with malignant melanoma.. Melanoma Res.

[50] Weterman MA, van Muijen GN, Bloemers HP, Ruiter DJ (1993). Expression of calcyclin in human melanocytic lesions.. Cancer Res.

[51] Zhou L, Beuerman R, Leonard A, Chan CM, Li SFY, Chew FT, Tan D (2009). Elevation of human α-defensins and S100 calcium binding protein A8 and A9 in tear fluid of pterygium patients.. Invest Ophthalmol Vis Sci.

